# Tactile and Proprioceptive Temporal Discrimination Are Impaired in Functional Tremor

**DOI:** 10.1371/journal.pone.0102328

**Published:** 2014-07-22

**Authors:** Michele Tinazzi, Alfonso Fasano, Alessia Peretti, Francesco Bove, Antonella Conte, Carlo Dall'Occhio, Carla Arbasino, Giovanni Defazio, Mirta Fiorio, Alfredo Berardelli

**Affiliations:** 1 Department of Neurological and Movement Sciences, University of Verona, Verona, Italy; 2 Division of Neurology, Toronto Western Hospital, University of Toronto, Toronto, Ontario, Canada; 3 Department of Neurology, Università Cattolica, Rome, Italy; 4 Department of Neurology and Psychiatry, Sapienza, University of Rome and IRCCS INM Neuromed, Pozzilli, Italy; 5 Division of Neurology, Ospedale di Voghera, Voghera, Italy; 6 Department of Basic Medical Sciences, Neuroscience and Sensory Organs, University of Bari, Bari, Italy; University of Ottawa, Canada

## Abstract

**Background and Methods:**

In order to obtain further information on the pathophysiology of functional tremor, we assessed tactile discrimination threshold and proprioceptive temporal discrimination motor threshold values in 11 patients with functional tremor, 11 age- and sex-matched patients with essential tremor and 13 healthy controls.

**Results:**

Tactile discrimination threshold in both the right and left side was significantly higher in patients with functional tremor than in the other groups. Proprioceptive temporal discrimination threshold for both right and left side was significantly higher in patients with functional and essential tremor than in healthy controls. No significant correlation between discrimination thresholds and duration or severity of tremor was found.

**Conclusions:**

Temporal processing of tactile and proprioceptive stimuli is impaired in patients with functional tremor. The mechanisms underlying this impaired somatosensory processing and possible ways to apply these findings clinically merit further research.

## Introduction

Patients with functional movement disorders may have a range of movement abnormalities including tremor.[1], [2] Although the clinical features in patients with functional movement disorders are well described, the pathophysiology of these conditions is largely unknown.[3] Over the years, neurophysiological and neuroimaging studies, have attempted to define a neurobiological model for some of these conditions.[4]–[6] Studies with transcranial magnetic stimulation are limited to patients with functional dystonia and demonstrated cortical and spinal inhibitory motor system abnormalities similar to those described in the organic forms.[4], [7] Unlike patients with primary dystonia, patients with functional dystonia nevertheless have normal sensorimotor cortical plasticity.[8]

We have previously found that the somatosensory processing underlying the ability to perceive two tactile stimuli as separate in time (TDT) was impaired in patients with functional dystonia.[9] Others reported an abnormal TDT in primary dystonias,[10], [11] in non-manifesting DYT1 mutation carriers,[12] in unaffected relatives,[13] and in Parkinson's disease.[14]–[16]

Another measure for assessing somatosensory processing is the proprioceptive temporal discrimination motor threshold (TDMT), defined as the shortest interval at which the subject perceives two externally-induced passive movements as separate in time.[17] The TDMT is normal in patients with focal dystonia and abnormal in those with essential tremor (ET).[18], [19] Because ET probably involves the cerebellar and brainstem oscillating loops,[20] an abnormal TDMT could depend on cerebellar dysfunction. Accordingly, TDMT testing disclosed abnormal findings also in patients with cerebellar degenerative ataxias.[21]

Given that previous imaging studies in patients with various functional movement disorders – including functional tremor (FT) – showed abnormal activation in both basal ganglia and cerebellum, as well as in several cortical areas,[5], [6], [22], [23] which are also involved in tactile and proprioceptive temporal discrimination,[24], [25] we conjectured that TDT and TDMT testing might disclose abnormal temporal processing in patients with FT. Therefore, we investigated TDT and TDMT in patients with FT, ET and healthy controls (HC).

## Methods

### Subjects

We recruited 11 right-handed patients with documented or clinical evidence of FT according to Fahn and Williams criteria,[26] as confirmed by two independent neurologist experts in movement disorders. Results were compared with those of a group of 13 age-matched normal control subjects and with those of 11 right-handed patients fulfilling diagnostic criteria for ET[27] (Table 1). The severity of arm tremor was rated by means of Tremor Rating Scale (TRS).[28] Subjects received detailed information about experimental procedures and provided written informed consent before attending to the study. No participant exhibited cognitive decline or any other limitation of the ability to provide an informed consent, therefore no next of kin or legally authorised representative was involed in this process. The ethical committee of Department of Neurological and Movement Sciences, University of Verona, Italy, approved the study.

**Table 1:** Demographic and clinical features in the patients with functional tremor (FT) and essential tremor (ET).

Group	N	Sex	Age	Tremor duration (yrs)	Tremor distribution	Other movement disorders	TRS	Management (daily dose mg)
FT	1	F	70	3	UL	None	21	Propranolol (40), Clonazepam (2.5)
FT	2	M	23	13	UL	None	26	Propranolol (40)
FT	3	F	44	14	UL,HEAD	Torticollis	13	Pregabalin (100), Clonazepam (2.5)
FT	4	F	60	2	UL	None	6	Venlafaxine (300)
FT	5	M	31	3	L UL	L UL dystonia	9	None
FT	6	F	63	8	UL	None	35	Primidone (250)
FT	7	M	59	6	UL,LL	None	36	Propanolol (40)
FT	8	M	45	7	L UL,L LL	L Knee buckling	7	Lorazepam(2.5)
FT	9	F	57	21	L UL, HEAD	None	5	Citalopram (10)
FT	10	M	23	5	R UL	None	1	None
FT	11	F	46	6	UL	None	8	None
**Mean±SD**		**6F/5M**	**47.4 ±16.2**	**8.0±5.8**			**15.0±12**	
ET	1	M	65	37	UL	None	15	None
ET	2	M	52	8	UL	None	8	Propanolol (40)
ET	3	F	68	41	UL	None	15	Bromazepam (0.5)
ET	4	M	54	6	UL	None	9	None
ET	5	M	58	7	UL	None	4	None
ET	6	F	59	5	UL, HEAD	None	35	Propanolol (40), Clonazapam (2.5)
ET	7	F	23	5	UL	None	14	Clonazepam (2.5)
ET	8	F	30	6	UL	None	21	Primidone (250)
ET	9	F	45	4	UL	None	10	Lorazepam (1)
ET	10	F	40	8	UL	None	26	Propanolol (40)
ET	11	M	43	5	UL	None	18	Propanolol (40)
**Mean±SD**		**6F/5M**	**48.9±14.3**	**12.0±13.4**			**15.8±9.0**	

Abbreviations: M: male; F: female; R: right, L: left; UL: upper limb(s); LL: lower limb(s); TRS: tremor rating score (see methods).

### Stimuli and procedure

Each upper limb was tested separately, and the order of presentation including the stimuli procedure was counterbalanced across subjects. To maintain subjects' attention throughout the procedure and to disclose possible perseverative responses, the ascending series for each procedure included catch trials (3 for each series) delivered at an interstimulus interval of 0 msec.

Tactile TDT testing was conducted according to previous standardized protocols.[9], [29] The value at which the subject recognized the two tactile stimuli as sequential for at least 3 consecutive intervals was defined as the TDT.

In order to assess TDMT, the first dorsal interosseous (FDI) and flexor carpi radialis (FCR) muscles were selectively stimulated with a procedure extensively described in previous studies.[17], [18] We defined the TDMT as the shortest interval elapsing between two paired electrical stimuli in which the subjects blindfolded perceived two separate index finger abductions (in response to FDI stimulation) and wrist flexions (in response to FCR stimulation) for at least three consecutive intervals.

Online Material S1 contains additional information on clinical features, neurophysiological procedures and statistical analysis.

## Results

No differences were found between the three groups of subjects (FT, ET, HC) for age or gender distribution. Severity (TRS scores – Table 1) and duration of tremor were comparable between patients with ET and those with FT (*p* = 0.554 and *p* = 0.667).

**Figure 1:**
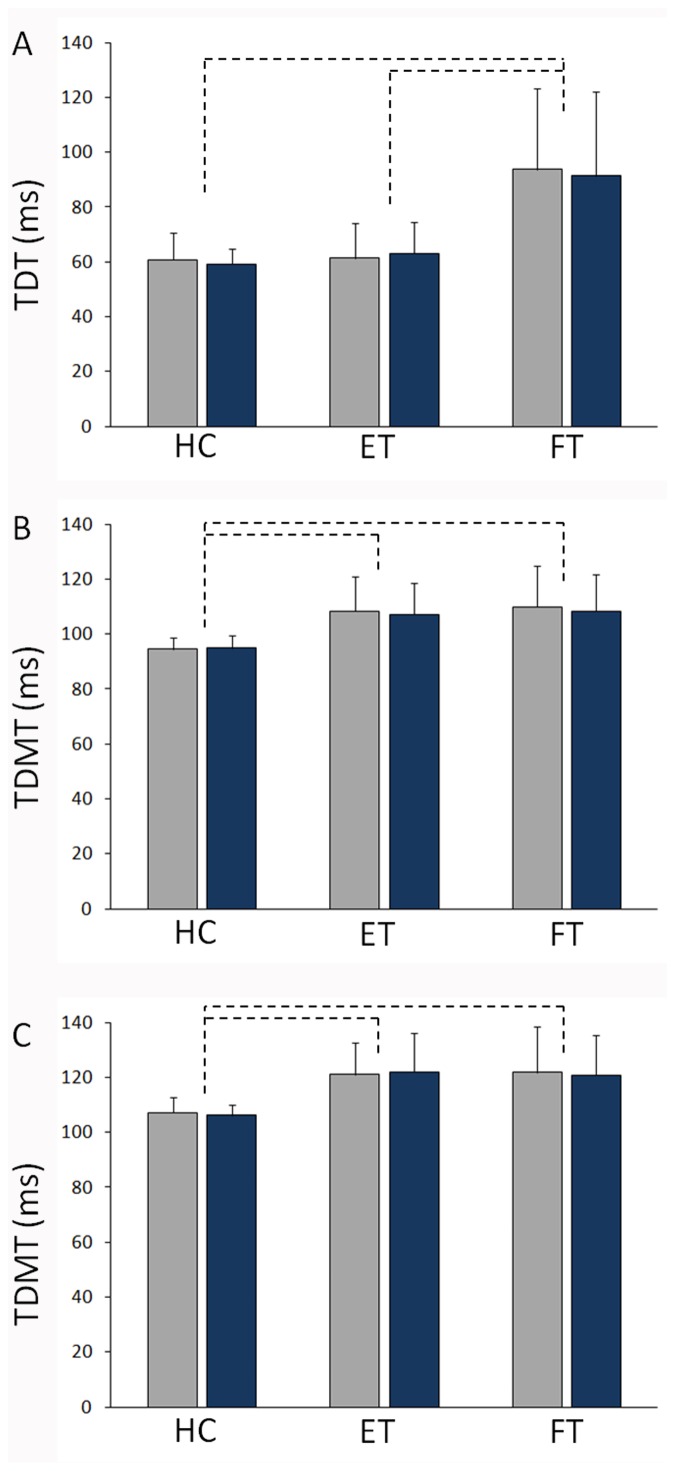
TDT and TDMT in patients with ET, FT and healthy controls. A) Tactile temporal discrimination thresholds (TDT) obtained in healthy controls (HC), patients with essential tremor (ET) and functional tremor (FT) after stimuli applied separately to the right (blue bars) and left (grey bars) hand; B) Temporal discrimination movement thresholds (TDMT) obtained in HC, and patients with ET, and FT after stimuli applied separately to the right (blue bars) and left (grey bars) first dorsal interosseous (FDI); C) and flexor carpii radialis (FCR) muscles.

TDT values in both right and left hands were higher in patients with FT than in those with ET and HC. Repeated-measures ANOVA identified a main effect of *Group* (F(2,32) = 11.5, *p*<0.001, *effect size* = 0.85), but no main effect of *Side* (F(1,32) = 0.14, *p* = 0.716, *effect size* = 0.06) or a *Side × Group* interaction (F(2,32) = 0.47, *p* = 0.632, *effect size* = 0.17) (Figure 1 A).

TDMT values in both the right and left FDI and FCR muscles were higher in patients with FT and ET than in HC. Repeated-measures ANOVA identified a main effect of *Group* (F(2,32) = 7.42, *p* = 0.002, *effect size* = 0.68), but no main effect of *Side* (F(1,32) = 0.33, *p* = 0.568, *effect size* = 0.10) or a *Side × Group* interaction (F(2,32) = 0.16, *p* = 0.852, *effect size* = 0.10) (Figure 1B and C). ANOVA also identified a main effect of muscle (F(1,32) = 380.9, p<0.001, *effect size* = 3.46) with higher TDMT values in FCR than in FDI muscle. The *Muscle × Group* (F(2,32) = 0.78, *p* = 0.468, *effect size* = 0.22) and the *Side × Muscle* (F(1,32) = 0.19, *p* = 0.670, *effect size* = 0.08) interactions were not significant. Nor was the triple interaction *Group × Side × Muscle* (F(2,32) = 1.34, *p* = 0.276, *effect size* = 0.29).

In the four FT patients with unilateral tremor (Patients n° 5, 8, 9, 10 of Table 1), TDT and TDMT values of the affected side were not significantly different from those of the unaffected side.

In patients with FT and ET Spearman rank correlation analysis disclosed no significant correlation between TDMT or TDT abnormalities and duration or severity of tremor.

To assess the reproducibility of TDT and TDMT measurements, the experiment was repeated in 5 patients with FT (patients n. 4–8 in Table 1) who accepted to be re-tested after a mean 10.4±2.2 months had elapsed. When comparing follow-up with baseline evaluation, tremor remained unchanged between and no difference was found in TDT values (right: 104±29 vs. 100±28.5; left: 107±28.2 vs. 112±24.9) (values expressed as ms±SD; for all comparisons, *p*>0.131). Nor did follow-up TDMT values for FDI or FCR muscle differ from baseline values (right FDI: 115.5±14.2 vs. 115.5±11.2; left: 117.5±17 vs. 114.5±16.6; right FCR: 132.5±7.5 vs. 130.5±12; left: 135±14.1 vs. 128±18.7) (values expressed as ms±SD; for all comparisons, *p*>0.144).

## Discussion

Our study shows that patients with FT have higher TDT and TDMT values than HC and ET patients hence FT patients have an impaired temporal processing of both tactile and proprioceptive stimuli. In ET we confirm our previous finding showing that the TDT is normal whereas the TDMT is increased compared to HC.[19]

The precise brain areas and circuits responsible for TDT changes remain unclear. Studies in healthy subjects attribute a crucial role in controlling the TDT to SI and pre-SMA cortical areas[25], [30] and investigations in patients with movement disorders or focal brain lesions suggest that abnormal TDT values reflect changes in basal ganglia and cerebellum.[14], [15], [21], [31], [32] Proprioceptive sensory information for the TDMT travels through the dorsal spinocerebellar tract and is processed through a distributed neural network in which the cerebellum and the parietal cortex play a prominent role.[33]–[35] Consistent with this hypothesis a functional MRI study in healthy subjects disclosed a larger number of voxels activated during electrically induced ankle dorsiflexion than in subjects at rest, and specifically contralateral SI and SII and cerebellum.[36] Overall studies in HC suggest that TDT and TMDT variables depend on cortico-subcortical circuits that only partially overlap.[37]

The abnormalities we found in TDT and TDMT in FT may be due to a specific impairment in attention.[38], [39] Although it is well known that FT disappears with distraction, we believe that an impaired attention is unlikely to play a role, given that tremor was absent during experiment (no patient had resting tremor). Nonetheless, patients with functional symptoms have a general increase in attention towards the body and new physical signs can easily be elicited during the examination by focusing attention onto the body. However, we believe that the impairment of the tactile and proprioceptive temporal discrimination seen in FT patients represents an intrinsic feature of FT. First, we tried to minimize the bias due to possible changes in attention by delivering catch trials (3 for each series) with 0 msec interstimulus interval, as already described in previous studies;[11], [15], [21] second, FT patients performed similarly on repeated tests, while we should have found a marked variability if these results would have been caused by attention deficits; third, we found a consistent bilateral pattern of impairment also in patients with unilateral symptoms; finally, abnormal temporal processing of somatosensory input in FT is in line with the abnormal TDT we reported in patients with functional dystonia,[9] although in that study we did not test the TDMT.

Functional neuroimaging studies performed during specific tasks in patients with functional movement disorders documented an abnormal activation in brain areas involved in motor planning and execution, including basal ganglia, cerebellum, parietal cortex and SMA, and in temporoparietal junction and limbic regions (insula, amygdala and cingulated cortex) involved in multisensory integration and motor prediction.[5], [6], [22], [23] Neurons in the insula respond to simple, innocuous, cutaneous stimuli[40] and integrate tactile, gustatory, olfactory, visual, auditory and visceral stimuli with emotional and attentional information.[41] Because tactile and proprioceptive afferent inputs processing requires a correct sensory integration in the limbic structures, dysfunction in the limbic circuits present in functional disorders[41] may explain the abnormalities in TDT and TDMT we observed. Abnormal activation in prefrontal regions, commonly reported in patients with functional motor symptoms,[42]–[44] could also alter the performance of self-report tasks including temporal discrimination.

Our study has a few limitations. The small sample size and cross-sectional design might have made our results difficult to interpret. Our study, nonetheless, compares favourably with the existing literature on functional neurological disorders, in which most studies have small sample sizes (from one to eight patients) due to the difficulties in enrolling these patients. For the same reason, we decided to characterize the patients only clinically without performing additional tests which would had required a multisession study design. Although a possible role played by psychotropic medications might be another confounding factor, our previous observations in organic tremor excludes this possibility because we did not find any effect of medication on TDT or TDMT. The strength of our study design is that we included patients with tremor who were clinically homogeneous and compared the results with those obtained not only in HC but also in patients with ET.

In conclusion, the finding that patients with FT have an abnormality of both TDT and TDMT (and not of one of the two, as seen in dystonia, ET and cerebellar degeneration) supports the hypothesis that in FT there is an unspecific impairment of sensory processing involving non-encoding neural structures. Future studies addressing whether patients with FT also present abnormalities in temporal processing of other sensory modalities would give further insight into the pathophysiology of FT.

## Supporting Information

Online Material S1 **Supporting Information.** Additional information on clinical features, neurophysiological procedures and statistical analysis.
(DOCX)
