# Correction: Tactile and Proprioceptive Temporal Discrimination Are Impaired in Functional Tremor

**DOI:** 10.1371/journal.pone.0111287

**Published:** 2014-10-10

**Authors:** The PLOS ONE Staff

The sixth author’s name is spelled incorrectly. The correct name is: Carlo Dallocchio.
