# Supplementary material for: Tactile and Proprioceptive Temporal Discrimination Are Impaired in Functional Tremor
Source: PLoS One. 2014 Jul 22;9(7):e102328. doi: 10.1371/journal.pone.0102328 (PMC4106827; doi:10.1371/journal.pone.0102328)
Supplement: Online Material S1 — Supporting Information. Additional information on clinical features, neurophysiological procedures and statistical analysis. (DOCX) [file pone.0102328.s001.docx]

**TACTILE AND PROPRIOCEPTIVE TEMPORAL DISCRIMINATION IN FUNCTIONAL TREMOR**

Michele Tinazzi^1^*, MD, PhD, Alfonso Fasano^2^*, MD, PhD, Alessia Peretti^1^, MD, Francesco Bove^4^, MD, Antonella Conte^4^, MD, PhD, Carlo Dall’Occhio^5^, Carla Arbasino^5^, MD, Gianni Defazio^6^, MD, Mirta Fiorio^1^, PhD, and Alfredo Berardelli^4^, MD

** contributed equally to this article*

^1^ Dipartimento di Scienze Neurologiche, Neuropsicologiche, Morfologiche e Motorie, Università di Verona, Italy

^2^ Division of Neurology, Toronto Western Hospital, University of Toronto, Toronto, Ontario, Canada

^3^ Dipartimento di Neurologia, Università Cattolica, Roma, Italy

^4^ Dipartimento di Neurologia e Psichiatria, Sapienza, Università di Roma and Istituto Neuromed, Pozzilli, Italy

^5^ Unità Operativa di Neurologia, Ospedale di Voghera, Italy

^6^ Dipartimento di Scienze Neurologiche e Psichiatriche, Università di Bari, Italy

**Online Material S1**

***Subjects***

All enrolled subjects underwent an extensive neurological examination in order to clinically exclude patients with somatosensory abnormalities and cognitive impairment (Mini-Mental State Examination > 26/30). Patients with resting tremor were also excluded from the analysis in order to avoid possible interferences of tremor with the TDT and TDMT measurement. In both groups, neurological dugs have been withdrawn one week before the assessment.

All subjects gave their written informed consent before participation. The study was approved by the institutional review boards at the recruiting center and conformed to the Declaration of Helsinki.

***Stimuli and procedure***

All participants were tested by a single neurologist expert in clinical neurophysiology (A.P) in a single experimental session lasting about 2 hours. Each upper limb was tested separately, and the order of presentation including the stimuli procedure was counterbalanced across subjects. Verbal instructions about the experimental tasks were also provided before each testing session, as well as examples of single or double stimuli. To maintain subjects’ attention throughout the procedure and to disclose possible perseverative responses, the ascending series for each procedure included catch trials (3 for each series) delivered at an interstimulus interval (ISI) of 0 msec. Subjects were allowed to pause between each block.

*Tactile temporal discrimination threshold (TDT)*

Tactile TDT testing was conducted according to previous standardized protocols.^1,2^ For each hand, we repeated the measurements 4 times and the 4 TDT values were then averaged and entered into the data analysis. Paired tactile stimuli consisted of square-wave electrical pulses delivered by a constant current stimulator through surface skin electrodes (1 mm in diameter) attached with adhesive pads to the index finger on the right or left hand. The anode was located 1.5 cm distally from the cathode. The intensity of tactile stimulation was determined for each subject by delivering a series of stimuli at increasing intensity (from 1 mA). Care was taken that stimuli induced no pain or discomfort. For each hand, combined stimuli were delivered in four separate blocks. Stimuli were delivered in pairs starting from simultaneous stimuli (ISI = 0 msec) and the ISI was progressively increased in 10 msec steps up to 300 msec. The value at which the subject recognized the two tactile stimuli as sequential for at least 3 consecutive intervals was defined as the TDT.

*Temporal discrimination movement threshold (TDMT)*

The first dorsal interosseous (FDI) and flexor carpi radialis (FCR) muscles were selectively stimulated with a procedure extensively described in previous studies.^3,4^ An insulated tungsten needle microelectrode (cathode) was inserted at a previously localized motor point (MP) in FDI and FCR muscles. The MP corresponds to the position in a muscle at which threshold for evoked contraction is minimal and from which stimuli delivered at 1 mA intensity elicit a clear twitch. The MP was first determined for each subject by surface stimulation through a probe electrode (2-mm diameter) in different positions over the muscle. During the experiment the microelectrode was inserted at the MP to provide stimulation. The anode was a surface electrode positioned 2–3 cm distally to the cathode. The stimulus (0.2 msec duration) was delivered at an intensity ranging from 1 to 2 mA and produced a painless twitch in the FDI muscle (causing an index finger abduction) and FCR muscle (causing a wrist flexion), without inducing radiating cutaneous paresthesias or sharp sensations. For each muscle, combined stimuli were delivered in four separate blocks. Before starting the task, subjects were trained to maintain the FDI and FCR muscles relaxed helped by EMG auditory feedback. When subjects were able to maintain the muscles relaxed, the testing session started. During the testing session, the subjects were blindfolded and wore ear-plugs to prevent visual and auditory feedback. Stimuli were delivered in pairs starting from simultaneous stimuli (ISI = 0 msec) and ISIs were progressively increased in 10 msec steps up to 300 msec. We defined the TDMT as the shortest interval elapsing between two paired electrical stimuli in which the subjects blindfolded perceived two separate index finger abductions (in response to FDI stimulation) and wrist flexions (in response to FCR stimulation) for at least three consecutive intervals. For each muscle, the values obtained from 4 measurements were averaged and entered in the data analysis.

To assess the reproducibility of TDT and TDMT measurements, the experiment was repeated in 5 patients with FT (patients n. 4-8 in Table 1) after a mean 10.4 ± 2.2 months had elapsed. In these patients tremor remained unchanged between the 2 assessments.

***Statistical analysis***

Data were expressed as mean ± SD unless otherwise indicated. Differences across groups were examined using Mann-Whitney U test, chi square test or Fisher’s test as appropriate, and one way ANOVA with post hoc paired t test. Separate analyses of variance (ANOVAs) were run to test TDT and TDMT values.

ANOVA for TDT had one between-subjects factor: Group (3 levels: FT, ET, HC) and one within-subjects factor: Stimulus side (2 levels: right, left). ANOVA for TDMT had one between-subjects factor: Group (3 levels: FT, ET, HC) and two within-subjects factor: Stimulus side (2 levels: right, left) and Muscle (2 levels: FDI, FCR). Post hoc comparisons with Bonferroni correction were used to check for differences among groups. Spearman’s rank-correlation analysis was used to test correlations between disease duration or disease severity (as assessed by TRS) of patients and TDT or TDMT values. Reproducibility of TDT and TDMT measurements in the 5 patients with FT was analysed by means of non-parametric Wilcoxon test by comparing the baseline and the follow-up values separately for the right and the left side and for the two muscles with regard to the TDMT. Significance level was set at *P*< 0.05.

**REFERENCES**

1. Morgante F, Tinazzi M, Squintani G, Martino D, Defazio G et al (2011) Abnormal tactile temporal discrimination in psychogenic dystonia. Neurology 77:1191-1197.
2. Fiorio M, Tinazzi M, Bertolasi L, Aglioti SM (2003) Temporal processing of visuotactile and tactile stimuli in writer's cramp. Ann Neurol 53:630-635.
3. Tinazzi M, Stanzani C, Fiorio M, Smania N, Moretto G et al (2005) Temporal discrimination of two passive movements in humans: a new psychophysical approach to assessing kinaesthesia. Exp Brain Res 166:184-189.
4. Tinazzi M, Fasano A, Di Matteo A, Ricciardi L, Bovi T et al (2013) Temporal discrimination in patients with dystonia and tremor and patients with essential tremor. Neurology 80:76-84.
